# Cellular oxygen consumption in patients with diabetic ketoacidosis

**DOI:** 10.1186/s40635-024-00673-0

**Published:** 2024-11-04

**Authors:** Jacob Vine, John H. Lee, Lakshman Balaji, Anne V. Grossestreuer, Andrea Morton, Natia Peradze, Nivedha Antony, Noa Berlin, Max S. Kravitz, Shannon B. Leland, Katherine Berg, Ari Moskowitz, Michael W. Donnino, Xiaowen Liu

**Affiliations:** 1 Center for Resuscitation Science, Beth Israel Deaconess Medical Center, 330 Brookline Ave, Boston, MA 02215 USA; 2 Department of Emergency Medicine, Beth Israel Deaconess Medical Center, 330 Brookline Ave, Boston, MA 02215 USA; 3 Department of Clinical Sciences, Cummings School of Veterinary Medicine at Tufts University, North Grafton, MA USA; 4 Department of Anesthesiology, Critical Care and Pain Medicine, Boston Children’s Hospital, Boston, MA USA; 5 Division of Pulmonary, Critical Care, and Sleep Medicine, Beth Israel Deaconess Medical Center, 330 Brookline Ave, Boston, MA 02215 USA; 6 Division of Critical Care Medicine, Montefiore Medical Center, The Bronx, NY USA; 7 Bronx Center for Critical Care Outcomes and Resuscitation Research, The Bronx, NY USA; 8 Present Address: Department of Microbiology and Pathology at Brigham Women’s Hospital, Boston, MA USA; 9 Present Address: Invicro, Needham, MA USA

**Keywords:** Diabetes, Diabetic ketoacidosis, Mitochondria, Thiamine, Coenzyme Q10, CoQ10

## Abstract

**Background:**

Diabetic ketoacidosis (DKA) is a potentially life-threatening disorder associated with severe alterations in metabolism and acid–base status. Mitochondrial dysfunction is associated with diabetes and its complications. Thiamine and coenzyme Q10 (CoQ10) are important factors in aerobic metabolism. In this study, we measured cellular oxygen consumption rates (OCRs) and the effects of in vitro administration of thiamine and CoQ10 on OCRs in patients with DKA versus healthy controls.

**Methods:**

Blood samples were collected from a prospective cohort of patients with DKA and from controls. Cellular OCRs were measured in peripheral blood mononuclear cells (PBMC) without treatment and after treatment with thiamine, CoQ10, or both. The mitochondrial profile was measured using an XFe96 Extracellular Flux Analyzer and XF Cell Mito Stress Test Kit (Seahorse Bioscience). A linear quantile mixed model was used to compare OCRs and estimate treatment effects.

**Results:**

A total of 62 patients with DKA and 48 controls were included in the study. The median basal and maximal OCRs were lower in the DKA group than in the control group (basal: 4.7 [IQR: 3.3, 7.9] vs. 7.9 [5.0, 9.5], *p* = 0.036; maximal: 16.4 [9.5, 28.1] vs. 31.5 [20.6, 46.0] pmol/min/µg protein, *p* < 0.001). In DKA samples, basal and maximal OCRs were significantly increased when treated with thiamine, CoQ10, or both. In controls, basal and maximal OCR were significantly increased only with thiamine treatment.

**Conclusion:**

Mitochondrial metabolic profiles of patients with DKA demonstrated lower cellular oxygen consumption when compared to healthy controls. Oxygen consumption increased significantly in cells of patients with DKA treated with thiamine or CoQ10. These results suggest that thiamine and CoQ10 could potentially have therapeutic benefits in DKA via their metabolic effects on mitochondrial cellular respiration.

## Background

Diabetic ketoacidosis (DKA) is a potentially life-threatening disorder associated with profound alterations in metabolism and acid–base status. Even after recovering from the acute illness, it can be associated with significant morbidity and an increased risk of long-term health issues [1]. Mitochondrial dysfunction has been associated with complications of diabetes, and decreased pyruvate dehydrogenase (PDH) activity has been demonstrated in patients with DKA [2–4].

Thiamine (vitamin B1) is an essential cofactor for PDH and alpha-ketoglutarate dehydrogenase, enzymes required for the process of aerobic metabolism. A deficiency in thiamine directly results in impairment of aerobic metabolism [5, 6]. Severe thiamine deficiency can result in Beriberi disease with cardiovascular collapse, encephalopathy, severe lactic acidosis, and potentially death [7, 8]. Previous studies have shown that thiamine is often depleted in patients with diabetes, potentially contributing to morbidity associated with DKA [9–11]. For example, in a study from 2014, Moskowitz et al. found that 25% of patients with DKA were thiamine deficient, and the deficiency was associated with increased severity of lactic acidosis, potentially caused by impaired aerobic metabolism in these patients [11].

Coenzyme Q10 (CoQ10) is a fat-soluble molecule and antioxidant that has a key role in the electron transport chain [12]. Previous studies have found alterations in the concentration and functionality of CoQ10 in diabetic states, potentially contributing to its pathogenesis, and exogenous supplementation of CoQ10 in patients with diabetes has been proposed to alleviate oxidative stress, restore mitochondrial function, and improve glycemic control [13, 14].

Given that both thiamine and CoQ10 play important roles in aerobic metabolism and prior studies demonstrating mitochondrial dysfunction in diabetes and its complications, we hypothesized that 1) aerobic respiration in peripheral mononuclear cells (PBMCs) from patients with DKA will be lower than that in healthy controls and 2) supplementation with thiamine and/or CoQ10 will improve aerobic respiration. To assess this, we measured cellular oxygen consumption rates (OCRs) in PBMCs from patients with DKA and healthy controls using an XFe96 Extracellular Flux Analyzer and XF Cell Mito Stress Test Kit (Seahorse Bioscience, North Billerica, Massachusetts, USA). First, we compared the OCRs of the DKA patients and healthy controls at baseline. Second, we compared the OCRs after the PBMCs received in vitro supplementation with thiamine and CoQ10.

## Methods

### Design and setting

This was a prospective observational study on patients enrolled in the Thiamine as Adjunctive Therapy in Diabetic Ketoacidosis Trial (DKAT) (NCT03717896) [15]. Patients were admitted with DKA after presenting to the Emergency Department (ED) of Beth Israel Deaconess Medical Center (BIDMC), an urban tertiary care center in Boston, Massachusetts, USA, between December 2018 and March 2023. Healthy controls were enrolled at the same time as the DKAT patients. Further details regarding the clinical trial can be found elsewhere [15]. This observational study utilized blood samples included at time of enrollment in the parent trial, before any study drug was administered. The study was approved by the local institutional review board (IRB) (protocol no. 2018P000475), and written informed consent was obtained from each patient prior to enrollment.

### Study population

Adult patients (age ≥ 18 years) who presented to the ED and were diagnosed with DKA (defined by bicarbonate ≤ 15 mEq/L, anion gap > 12 mEq/L, pH ≤ 7.24, and urine or serum ketones ≥ 3 mmol/L) were included and enrolled within six hours of presentation [15]. Patients who received thiamine supplementation prior to enrollment; had a competing cause of severe acidosis (i.e., seizures, carbon monoxide poisoning, cyanide toxicity, or cardiac arrest); had a known allergy to thiamine; had contraindications to thiamine as determined by the clinical team; were a member of a research-protected population; and those who arrived with a do-not-resuscitate status were excluded. The control group included healthy individuals without any acute illnesses. Controls and DKA patients were enrolled concurrently to allow for simultaneous running of the mitochondrial test and minimization of measurement error. Therefore, many of the controls consisted of emergency department staff and providers and a control individual was allowed to be enrolled more than once at different time points.

### Data collection and blood samples

Baseline demographic data and laboratory values were collected by trained research assistants and entered into a secure online database [16]. Data items included demographics, medical history, and laboratory values. Blood samples were collected upon enrollment into the study prior to study-drug administration. Fresh whole blood was used to isolate PBMCs to measure the cellular OCR.

### Mitochondrial function analysis

PBMCs were isolated from fresh whole blood samples using the following method. Plasma was separated by centrifugation at 800 g for 15 min at 4 °C and saved without disturbing the buffy coat. Plasma was then replaced with the same volume of Roswell Park Memorial Institute Medium (RPMI), and the cell pellets were mixed by gently pipetting up and down several times to disperse the cells. PBMCs were then isolated from the RPMI substituted blood samples using a density gradient separation method according to the manufacture’s instruction (Ficoll-Paque premium; GE Healthcare Bio-Science Corporation, Piscataway, New Jersey, USA). The PBMCs were divided into four groups: (1) no treatment, (2) treatment with thiamine (0.5 μg/mL), (3) treatment with ubiquinol (reduced Coenzyme Q10, (CoQ10) (1 µg/mL), and (4) treatment with a combination of both thiamine and CoQ10. The dosing of thiamine and CoQ10, as well as the details of the experimental design, were based on our previous studies [17, 18]. The mitochondrial profile was measured using an XFe96 Extracellular Flux Analyzer and XF Cell Mito Stress Test Kit (Seahorse Bioscience, North Billerica, Massachusetts, USA). The XF Cell Mito Stress Test uses modulators of respiration that target components of the electron transport chain to measure key parameters of metabolic function. Oligomycin, carbonyl (cyanide-4-trifluoromethoxy) phenylhydrazone (FCCP), and a mix of rotenone and antimycin A were sequentially injected to inhibit specific components of the electron transport chain and provide isolated measurements of OCR for basal, maximal, ATP-linked, and non-mitochondrial respiration. From these measurements, spare respiratory capacity and proton-leaked respiration were calculated. The reported OCRs are in pmol/min/μg of protein. The protein amount was measured using the bicinchoninic acid (BCA) assay after lysing the cells. The experiment protocol followed the recommendations of the instrumentation company. Additional details regarding the instrument have been described elsewhere [17–19].

### Statistical analysis

Statistical analyses were performed using R software (R version 4.1.1) [20]. Baseline characteristics of categorical variables are presented as counts and proportions. For continuous variables, medians and interquartile ranges or means and standard deviations were used as appropriate based on the distribution of the data. The outcome OCR variables were summarized for each group and treatment using raw medians and interquartile ranges. To assess median differences, linear quantile mixed models (LQMM, Geraci 2014) [21] were used with a compound symmetry structure to account for within-patient clustering (as some samples originated from the same control enrolled at different times). We assessed (1) the median differences between DKA patients and healthy controls at baseline (without treatment), (2) the relative median differences between no treatment and treatment within each group, and (3) the effects of treatment in the DKA group compared to the same effect in the control group. For the first assessment, the LQMM included a single term for the group as the predictor variable, while for the latter two assessments, the LQMM included an interaction term between the group and treatment. In all models, the effect of age was controlled for by adding the logarithm of age as a covariate, given the potential effect of age on OCR. The relative median differences in OCR values for a given treatment were expressed as a proportion of the value at baseline (no treatment). Statistical significance was a priori set at *p* < 0.05.

## Results

A total of 110 patients (with 62 DKA patients and 48 healthy controls) were included in the study. The baseline characteristics of the study participants are summarized in Table 1.

**Table 1:** Baseline characteristics

Characteristic	DKA (*n* = 62)	Controls (*n* = 48)
Age (years, median, IQR)^a^	45 (27, 58)	24 (23, 28)
Male (*n*, %)	33 (53.2)	10 (20.8)
*Race* (*n*, %)		
White Black Asian Other Unknown	37 (59.7) 18 (29.0) 3 (4.8) 1 (1.6) 3 (4.8)	42 (87.5) 1 (2.1) 1 (2.1) 3 (6.3) 1 (2.1)
*Ethnicity* (*n*, %)		
Not Hispanic Hispanic Unknown	51 (82.3) 6 (9.7) 5 (8.1)	38 (79.2) 8 (16.7) 2 (4.2)
Diabetes (n, %)	62 (100)	2 (4.2)
Type I Type II Type III	51 (80.6) 10 (14.5) 1 (4.8)	2 (4.2) 0 0
*DKA history* (*n*, %)^b^		
Previous DKA No previous DKA Unknown	30 (48.4) 29 (46.8) 3 (4.8)	– – 2 (4.2)
*Other past medical history* (*n*, %)^c^		
CAD	5 (8.1)	0 (0.0)
Cancer	5 (8.1)	1 (2.2)
CHF	2 (3.2)	0 (0.0)
COPD	0 (0.0)	1 (2.2)
Alcohol use disorder	0 (0.0)	1 (2.2)
HIV/AIDS	1 (1.6)	0 (0.0)
Liver Disease	0 (0.0)	0 (0.0)
CKD	6 (9.7)	0 (0.0)
Stroke/TIA	1 (1.6)	0 (0.0)
History of tobacco use	5 (8.1)	0 (0.0)
Transplant/immunocompromised	0 (0.0)	0 (0.0)

^a^Age missing for one control patient. ^b^Diabetic ketoacidosis history unavailable for control patients. ^c^Past medical history missing for one control patient. *BMI* body mass index, *DKA* diabetic ketoacidosis, *CAD* coronary artery disease, *CHF* congestive heart failure, *COPD* chronic obstructive pulmonary disease, *HIV* human immunodeficiency virus, *AIDS* acquired immunodeficiency syndrome, *TIA* transient ischemic attack, *SOFA* sequential organ failure assessment

### Basal respiration

**Fig. 1:**
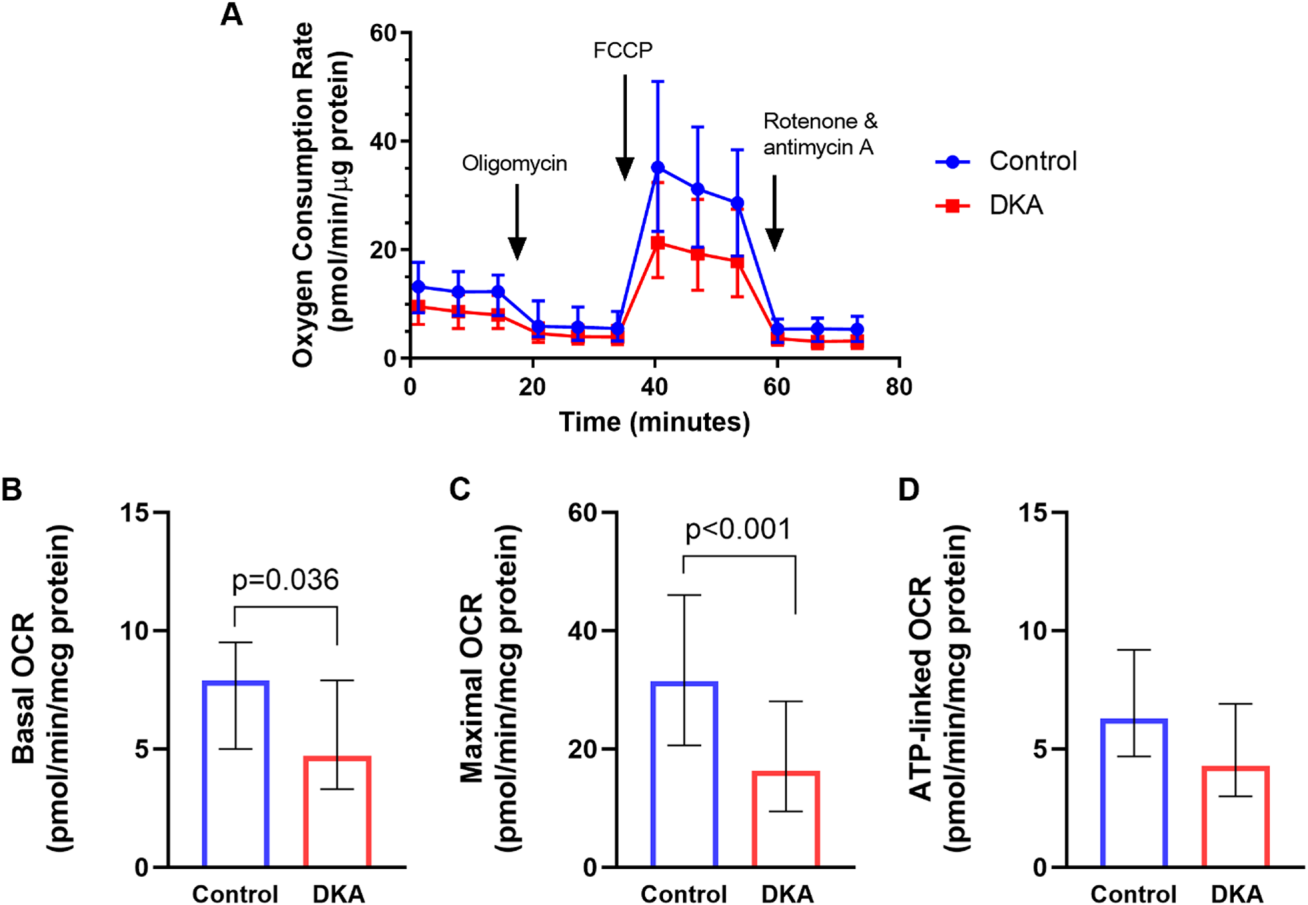
**A** Mitochondrial respiration profile demonstrating parameters of mitochondrial function in the DKA and control groups. **B** Comparison of baseline (no treatment) OCR for **B** Basal, **C** Maximal, and **D** ATP-linked. For both basal and maximal OCR, the DKA group had significantly lower OCR compared to the control group

**Fig. 2:**
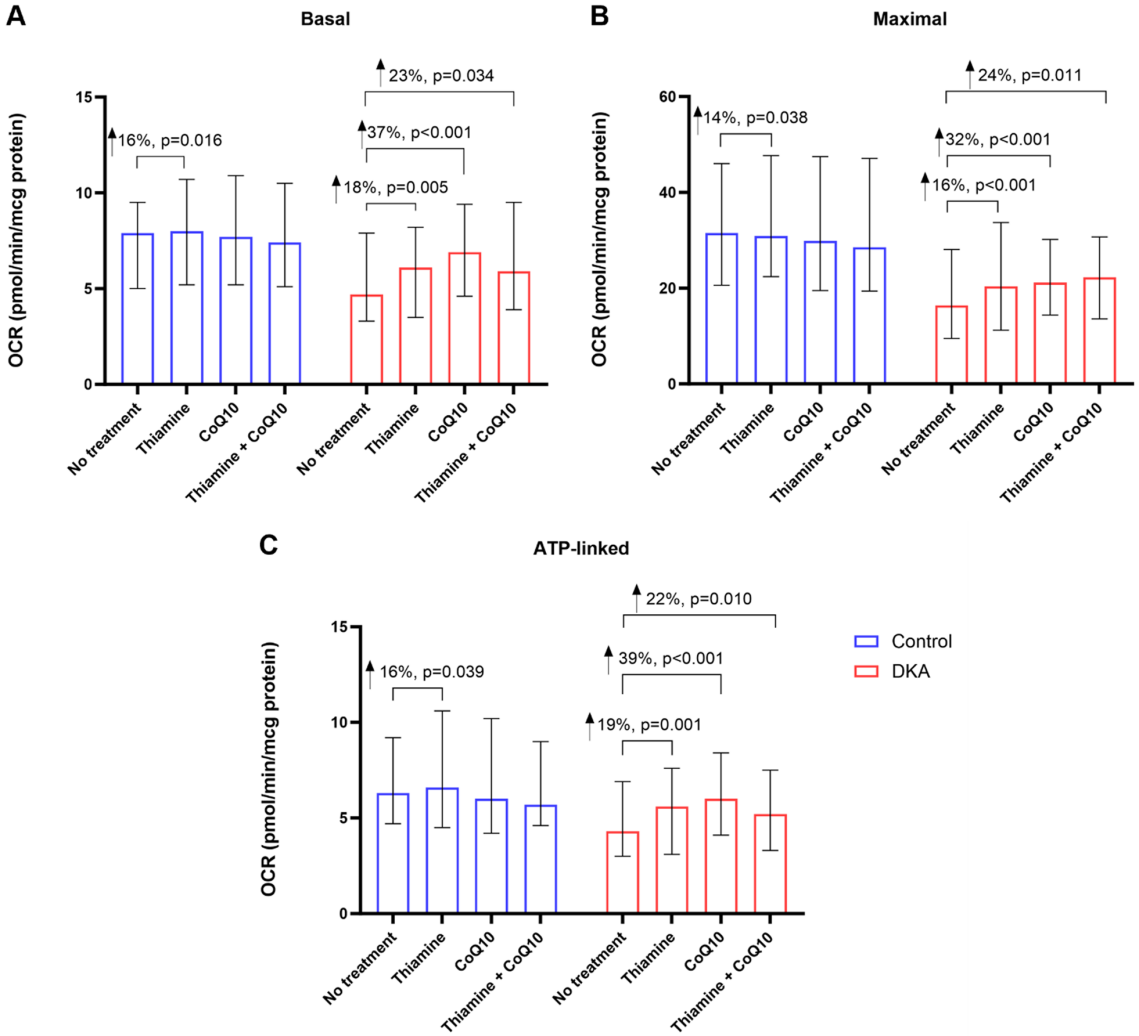
Comparison of OCR after treatment between DKA and control groups for **A** Basal, **B** Maximal, and **C** ATP-linked. Changes in OCR after treatment are noted in relation to the baseline OCR (no treatment) for each group

The baseline (no treatment) median basal OCR was significantly lower in DKA samples compared to controls (4.7 [3.3, 7.9] vs. 7.9 [5.0, 9.5] pmol/min/μg of protein) (Fig. 1B). With treatment, in DKA samples, there was significant increase in OCRs with all treatments. In the control samples, there was a significant increase in OCR only with thiamine. No significant increase was observed after treatment with CoQ10 or both thiamine and CoQ10 (Fig. 2A).

### Maximal respiration

The baseline median maximal OCR was also significantly lower in DKA samples compared to controls (16.4 [9.5, 28.1] vs. 31.5 [20.6, 46.0] pmol/min/μg of protein) (Fig. 1C) With treatment, in DKA samples, there was a significant increase in OCRs with all treatments. In the control samples, there was a significant increase in OCR only when treated with thiamine. No significant increase was observed after treatment with CoQ10 or both thiamine and CoQ10 (Fig. 2B).

### ATP-linked respiration

The baseline median ATP-linked OCR was not significantly different between DKA and controls (4.3 [3.0, 6.9] vs. 6.3 [4.7, 9.2] pmol/min/μg of protein) (Fig. 1D). With treatment, in DKA samples, there was a significant increase in OCRs with all treatments. In the control samples, there was a significant increase in OCR only when treated with thiamine (Fig. 2C). Additional measurements are presented in Tables 2 and 3.

**Table 2:** Comparison of Baseline (no treatment) OCR between DKA and control groups

	DKA (*n* = 62)	Control (*n* = 62)	Median difference (95% CI)	*p*-value
Basal	4.7 (3.3, 7.9)	7.9 (5.0, 9.5)	− 2.31 (− 4.47, − 0.15)	0.036
Maximal	16.4 (9.5, 28.1)	31.5 (20.6, 46.0)	− 14.58 (− 20.33, − 8.84)	< 0.001
ATP-linked	4.3 (3.0, 6.9)	6.3 (4.7, 9.2)	− 1.60 (− 3.65, 0.45)	0.124
Spare	11.4 (6.1, 21.1)	24.9 (13.4, 34.5)	− 12.41 (− 17.39, − 7.43)	< 0.001
Proton leak^a^	0.6 (0.0, 1.1)	0.8 (0.0, 1.7)	− 0.076 (− 0.80, 0.65)	0.834
Non-Mitochondrial^b^	2.9 (1.7, 4.2)	5.1 (2.6, 6.3)	− 1.79 (− 2.68, − 0.90)	< 0.001

*n* refers to the number of samples in each group. For the control group, the number of samples (62) was different from the number of patients, since some patients provided samples more than once at different time points. ^a^*n* = 58 (DKAT), *n* = 59 (Control). ^b^*n* = 57 (DKAT), *n* = 57 (Control). OCR is expressed as pmol/min/µg protein and is reported as medians (interquartile range). *DKA* diabetic ketoacidosis, *CI* confidence interval, *ATP* adenosine triphosphate

**Table 3:** Comparison of OCR with treatment

		DKA		Control	
Treatment		OCR (Median, IQR)	Relative median difference (%, 95% CI)	OCR (Median, IQR)	Relative median difference (%, 95% CI)
Basal	None	4.7 (3.3, 7.9), *n* = 62	–	7.9 (5.0, 9.5), *n* = 62	–
	Thiamine	6.1 (3.5, 8.2), *n* = 61	18 (6, 30), *p* = 0.005	8.0 (5.2, 10.7), *n* = 60	16 (3, 29), *p* = 0.016
	CoQ10	6.9 (4.6, 9.4), *n* = 60	37 (19, 55), *p* < 0.001	7.7 (5.2, 10.9), *n* = 59	11 (− 2, 24), *p* = 0.106
	Thiamine + CoQ10	5.9 (3.9, 9.5), *n* = 61	23 (2, 44), *p* = 0.034	7.4 (5.1, 10.5), *n* = 62	14 (− 1, 30), *p* = 0.064
Maximal	None	16.4 (9.5, 28.1), *n* = 62	–	31.5 (20.6, 46.0), *n* = 62	–
	Thiamine	20.4 (11.2, 33.7), *n* = 61	16 (8, 24), *p* < 0.001	30.9 (22.4, 47.7), *n* = 60	14 (1, 28), *p* = 0.038
	CoQ10	21.2 (14.4, 30.2), *n* = 60	32 (20, 45), *p* < 0.001	29.9 (19.5, 47.5), *n* = 58	− 6 (− 17, 6), *p* = 0.356
	Thiamine + CoQ10	22.3 (13.6, 30.7), *n* = 61	24 (6, 42), *p* = 0.011	28.5 (19.4, 47.1), *n* = 62	3 (− 13, 19), *p* = 0.704
ATP-linked	None	4.3 (3.0, 6.9), *n* = 61	–	6.3 (4.7, 9.2), *n* = 62	–
	Thiamine	5.6 (3.1, 7.6), *n* = 60	19 (8, 30), *p* = 0.001	6.6 (4.5, 10.6), *n* = 60	16 (1, 31), *p* = 0.039
	CoQ10	6.0 (4.1, 8.4), *n* = 58	39 (27, 51), *p* < 0.001	6.0 (4.2, 10.2), *n* = 58	1 (− 12, 13), *p* = 0.918
	Thiamine + CoQ10	5.2 (3.3, 7.5), *n* = 58	22 (6, 39), *p* = 0.010	5.7 (4.6, 9.0), *n* = 61	0.4 (− 11, 12), *p* = 0.938
Spare	None	11.4 (6.1, 21.1), *n* = 62	–	24.9 (13.4, 34.5), *n* = 62	–
	Thiamine	13.6 (6.6, 24.3), *n* = 61	21 (11, 31), *p* < 0.001	23.7 (12.8, 38.7), *n* = 60	16 (5, 28), *p* = 0.006
	CoQ10	14.9 (9.4, 22.6), *n* = 60	46 (27, 65), *p* < 0.001	22.6 (10.2, 36.4), *n* = 58	− 8 (− 19, 3), *p* = 0.146
	Thiamine + CoQ10	14.5 (8.5, 24.8), *n* = 61	39 (17, 61), *p* = 0.001	22.2 (12.7, 35.2), *n* = 62	1 (− 11, 14), *p* = 0.847
Proton leak	None	0.6 (0.0, 1.1), *n* = 58	–	0.8 (0.0, 1.7), *n* = 59	–
	Thiamine	0.7 (0.3, 1.4), *n* = 58	16 (– + 1223, 1254), *p* = 0.980	0.8 (0.0, 1.9), *n* = 56	− 350 (− 973, 272), *p* = 0.264
	CoQ10	0.9 (0.0, 1.8), *n* = 57	186 (− 511, 884), *p* = 0.594	1.0 (0.4, 2.4), *n* = 55	− 12 (− 228, 204), *p* = 0.914
	Thiamine + CoQ10	0.5 (0.0, 1.4), *n* = 57	52 (− 450, 554), *p* = 0.836	1.0 (0.3, 2.1), *n* = 59	− 15 (− 520, 489), *p* = 0.951
Non-Mito	None	2.9 (1.7, 4.2), *n* = 57	–	5.1 (2.6, 6.3), *n* = 57	–
	Thiamine	3.6 (2.7, 4.4), *n* = 57	26 (10, 42), *p* = 0.002	3.8 (2.5, 5.0), *n* = 56	− 9 (− 27, 10), *p* = 0.350
	CoQ10	3.9 (2.3, 5.3), *n* = 55	45 (23, 68), *p* < 0.001	4.1 (2.6, 6.0), *n* = 54	− 2 (− 14, 10), *p* = 0.741
	Thiamine + CoQ10	4.5 (2.9, 6.0), *n* = 57	53 (26, 80), *p* < 0.001	4.5 (2.8, 5.8), *n* = 58	7 (− 16, 29), *p* = 0.551

OCRs are reported as medians (interquartile ranges). *n* refers to the number of samples in each group. Relative median difference is the percentage of change relative to baseline values. *IQR* interquartile range, *DKA* diabetic ketoacidosis, *OCR* oxygen consumption rate, *Non-mito* non-mitochondrial

### Differential treatment effect

The effect of CoQ10 treatment on basal, maximal, and ATP-linked respiration was significantly greater in samples from patients with DKA than in those from controls. This differential effect was not significant in samples treated with thiamine or the combination of thiamine and CoQ10. Additional results of the interaction analysis are shown in Table 4.

**Table 4:** Analysis of interaction between DKA and treatment

Treatment		Relative median difference (%, 95% CI)	*p*-value
Basal	Thiamine	− 0.1 (− 17, 17)	0.987
	CoQ10	24 (4, 45)	0.020
	Thiamine + CoQ10	3 (− 23, 29)	0.802
Maximal	Thiamine	0.1 (− 17, 18)	0.991
	CoQ10	33 (15, 52)	0.001
	Thiamine + CoQ10	15 (− 14, 44)	0.295
ATP-linked	Thiamine	1 (− 17, 20)	0.905
	CoQ10	36 (21, 50)	< 0.001
	Thiamine + CoQ10	16 (− 5, 37)	0.127
Spare	Thiamine	4 (− 12, 21)	0.578
	CoQ10	54 (32, 76)	< 0.001
	Thiamine + CoQ10	37 (12, 62)	0.004
Proton leak	Thiamine	380 (− 522, 1282)	0.401
	CoQ10	28 (− 393, 449)	0.894
	Thiamine + CoQ10	29 (− 574, 633)	0.923
Non-Mito	Thiamine	33 (9, 57)	0.007
	CoQ10	46 (22, 69)	< 0.001
	Thiamine + CoQ10	44 (4, 83)	0.033

The relative median difference is the percentage of change relative to baseline values. CoQ10: Coenzyme Q10; ATP, adenosine triphosphate; Non-mito non-mitochondrial

## Discussion

In this study, we found that patients with DKA had impaired mitochondrial function in PMBCs with significantly lower basal, maximal, and ATP-linked respiration compared to healthy controls. We also examined the effect of in vitro thiamine and CoQ10 supplementation on cellular respiration and found that the treatment was generally associated with an increase in respiration. Specifically, in DKA samples, treatment with either thiamine, CoQ10, or both significantly increased basal, maximal, and ATP-linked OCRs. In the control group, only thiamine treatment was associated with a significant increase in OCRs.

The overall lower baseline OCRs observed in DKA samples compared to healthy controls are consistent with the hypothesis that mitochondrial function is impaired in DKA. The increase in OCRs observed with thiamine and CoQ10 supplementation in DKA samples provides evidence supporting the hypothesis that treatment with thiamine and CoQ10 could potentially improve mitochondrial function in DKA.

CoQ10 treatment increased the OCR in DKA samples to a greater extent than in the controls, and this differential effect was statistically significant. Thiamine treatment increased basal, maximal, and ATP-linked OCRs in both DKA samples and controls. However, there was no differential effect when comparing the effects of thiamine with controls. The underlying reason for the increase in OCR, also seen in controls, is unclear at this time but likely indicates that thiamine can increase the efficiency of aerobic metabolism to some degree, even without deficiency. The differential effect seen in CoQ10 treatment suggests that CoQ10 acts more specifically in the context of DKA, whereas its effect is lower in controls. Further studies are needed to better understand the underlying biochemical mechanisms and the differential treatment effects of CoQ10 compared to thiamine.

Mitochondrial dysfunction has been identified as a potential contributor to the pathophysiology of diabetes and its related complications. Mitochondria generate energy for cellular activities via oxidative phosphorylation through various pathways. Dysfunction of these pathways leads to widespread consequences, including impairment in energy production and generation of reactive oxygen species, which have been linked to immune-mediated destruction that characterizes type 1 diabetes, as well as pancreatic islet β-cell dysfunction and insulin resistance in type 2 diabetes [2, 3]. Given that thiamine and CoQ10 are important cofactors required for cellular respiration, deficiencies in these cofactors may contribute to impairment of aerobic respiration and mitochondrial dysfunction in DKA.

This study showed significant improvement in OCR with thiamine and CoQ10 supplementation in PMBCs of patients with DKA. To our knowledge, this is the first study to evaluate changes in mitochondrial respiration with in vitro thiamine and CoQ10 supplementation. These findings provide further evidence to support the hypothesis that (1) mitochondrial function and aerobic respiration are depressed in DKA, and (2) aerobic respiration can potentially be improved by treatment with thiamine and CoQ10. Although these findings are promising, it should be noted that this was a preliminary study and hypothesis generating.

The results of this study should be interpreted within the context of several limitations. First, this study evaluated in vitro thiamine and CoQ10 treatment and, therefore, further studies are needed to assess their effects in vivo. Second, OCRs were measured in PBMCs, and whether the findings will translate to other cell types is unclear. A randomized clinical trial of thiamine administration in DKA is currently ongoing, and may answer some of these questions, including exploring the effect of intravenous thiamine on cellular OCR (ClinicalTrial.Gov, NCT03717896). Third, we did not measure baseline thiamine and CoQ10 levels in the context of this study; therefore, we do not know if the treatment effect is due to deficiencies in these substrates or due to the metabolic cellular environment in the setting of DKA.

## Conclusion

Mitochondrial metabolic profiles of patients with DKA demonstrated lower cellular oxygen consumption when compared to healthy controls. Oxygen consumption increased significantly in cells of patients with DKA treated with thiamine and/or CoQ10. These results suggest that thiamine and CoQ10 could potentially have therapeutic benefits in DKA via their metabolic effects on mitochondrial cellular respiration.

## Data Availability

The datasets used and/or analyzed during the current study are available from the corresponding author on reasonable request.

## Abbreviations

ATP: Adenosine triphosphate
CoQ10: Coenzyme Q10
DKA: Diabetic ketoacidosis
IRB: Institutional review board
LQMM: Linear quantile mixed model
OCR: Oxygen consumption rate
PBMC: Peripheral blood mononuclear cell
PDH: Pyruvate dehydrogenase
